# Satellite-based characterization of climatic conditions before large-scale general flowering events in Peninsular Malaysia

**DOI:** 10.1038/srep32329

**Published:** 2016-08-26

**Authors:** Muna Maryam Azmy, Mazlan Hashim, Shinya Numata, Tetsuro Hosaka, Nur Supardi Md. Noor, Christine Fletcher

**Affiliations:** 1 Department of Tourism Science, Tokyo Metropolitan University, Hachiouji, 192-0397 Tokyo, Japan; 2 Geosciences & Digital Earth Centre (INSTeG), Research Institute of Sustainable Environment (RISE), Universiti Teknologi Malaysia, 81310 UTM Johor Bahru, Malaysia; 3 Faculty of Geoinformation & Real Estate, Universiti Teknologi Malaysia, 81310 UTM Johor Bahru, Malaysia; 4 Forestry Division, Forest Research Institute Malaysia, 52109 Kepong, Selangor, Malaysia

## Abstract

General flowering (GF) is a unique phenomenon wherein, at irregular intervals, taxonomically diverse trees in Southeast Asian dipterocarp forests synchronize their reproduction at the community level. Triggers of GF, including drought and low minimum temperatures a few months previously has been limitedly observed across large regional scales due to lack of meteorological stations. Here, we aim to identify the climatic conditions that trigger large-scale GF in Peninsular Malaysia using satellite sensors, Tropical Rainfall Measuring Mission (TRMM) and Moderate Resolution Imaging Spectroradiometer (MODIS), to evaluate the climatic conditions of focal forests. We observed antecedent drought, low temperature and high photosynthetic radiation conditions before large-scale GF events, suggesting that large-scale GF events could be triggered by these factors. In contrast, we found higher-magnitude GF in forests where lower precipitation preceded large-scale GF events. GF magnitude was also negatively influenced by land surface temperature (LST) for a large-scale GF event. Therefore, we suggest that spatial extent of drought may be related to that of GF forests, and that the spatial pattern of LST may be related to that of GF occurrence. With significant new findings and other results that were consistent with previous research we clarified complicated environmental correlates with the GF phenomenon.

The synchronized reproduction of Dipterocarpaceae and other families in aseasonal Southeast Asian rainforests at irregular intervals of 1–10 years is known as general flowering (GF)^1^,^2^,^3^,^4^,^5^,^6^. Some species, for example in Borneo^3^,^4^,^5^,^6^,^7^, reproduce only during GF events. General flowering results in massive fruit production and seedling establishment, in turn leading to the regeneration of dipterocarp forests in the region. Unlike other equatorial rainforests where synchronized flowering occurs at nearly the same time each year^8^, GF in Southeast Asian rainforests is unpredictable both spatially and temporally^2^,^5^,^9^.

Since various plant taxa synchronize their reproduction at irregular intervals and display similar flowering sequences in different GF events, they were assumed to respond to common triggers^2^. Several environmental cues have been proposed as triggering factors, including radiation period^10^, low night temperature^2^,^4^, and abnormally dry weather^2^,^4^,^5^,^6^,^11^,^12^,^13^. The relationship between drought and flowering as a possible trigger of GF is well known based on a study from Sarawak, Malaysia^12^ among others^1^,^6^,^14^,^15^. Identifying conditions that enhance GF occurrence is especially important as phenological reproductive events like GF can fluctuate due to droughts, which are often related to the broader effects of climate change.

The distribution of GF occurrence varies greatly over the landscape, while synchronization of this community-level phenomenon occurs among forests both within and among regions^16^,^17^. However, observations of climatic GF triggers at large spatial scales are limited because many studies have examined only single study sites, with poor spatial representation in terms of meteorological stations. As an exception, one study reported on variations in GF episodes at the regional scale by comparing three GF episodes from 2001–2005 in 10 states in Peninsular Malaysia, demonstrating that prolonged drought influenced only some aspects of GF intensity^6^. However, this conclusion relied on extrapolating data for multiple points from relatively few meteorological stations.

Spatio-temporal observations using remote sensing have increasingly been applied in environmental pattern assessment due to their affordability and the accessibility of current and archived data. Satellites continuously provide new data, such as above- and below-canopy measurements and radiometric information, and help to elucidate the phenology of forest canopies based on measureable biophysical quantities^18^. Remote sensing also provides more data than traditional field surveys, which facilitates understanding in ecological studies^19^.

Therefore, this study attempts to improve the use of meteorological data to describe external climatic cues before large-scale GF events using a remote sensing approach. We examined the potential climatic triggers proposed in previous studies, such as drought, radiation, and temperature, to investigate climatic anomalies before GF events over 10 years in an average of 75 forests on Peninsular Malaysia. To observe these climatic conditions, we used data from two satellite sensors: (a) the Tropical Precipitation Measuring Mission (TRMM), an advanced precipitation observation instrument used in climatological studies and applied research such as hydrological modeling^20^, and (b) the Terra satellite with Moderate Resolution Imaging Spectroradiometer (MODIS) sensor, which offers unlimited observations for atmospheric, terrestrial, and ocean phenomenology^21^. We took three satellite-derived estimates from these satellites—land surface temperature (LST)^22^, the fraction of photosynthetically active radiation (fPAR)^23^, and precipitation—as important indicators potentially describing the climatic conditions of the observed areas before GF events.

Recognizing potential climatic effects on GF is vital due to the major impact of GF on Southeast Asian rainforest regeneration. Several studies have highlighted the importance of understanding GF, and especially whether its frequency may be altered by climate change^3^,^6^,^24^. Our study aims to identify the climatic cues that trigger large-scale GF events in Peninsular Malaysia to determine where and when GF can be predicted by remote-sensing. We assumed that spatial and temporal patterns of climatic cue fluctuations are consistent with GF occurrences if GF forests share common climatic cues for synchronization^6^. We hypothesized that: (i) different antecedent climatic conditions are observed between large- and local-scale GF events temporally, and ii) that LST and fPAR before large-scale GF would be higher than those before local-scale GF while rainfall would be lower. If hypothesis (i) is supported, would the magnitude of preceding climatic conditions spatially affect the magnitude of large-scale GF? This comparison of climatic conditions among large-scale GF events using remote sensing data will help to reveal the external spatiotemporal drivers of large-scale GF events.

## Results

### GF magnitude over 10 years

**Figure 1:**
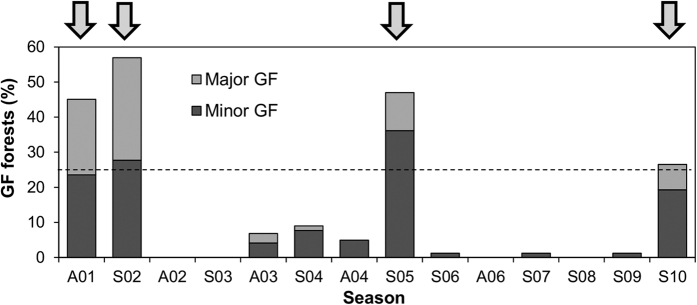
Percentage of GF forests in 14 observations (refer to Table S1 for GF codes). The dashed line at 25% fruiting magnitude is a threshold indicating large-scale GF events (arrows).

Based on GF scores, we found four large-scale GF events over 10 years: one autumn-type event in 2001 and three spring-type events in 2002, 2005, and 2010 (Fig. 1). The other events were classified as local-scale GFs. However, autumn-type GF occurrences could not be assessed from 2005 to 2010, with the exception of 2006, due to a lack of GF survey data.

### Antecedent climatic conditions of large and local-scale GF

**Figure 2:**
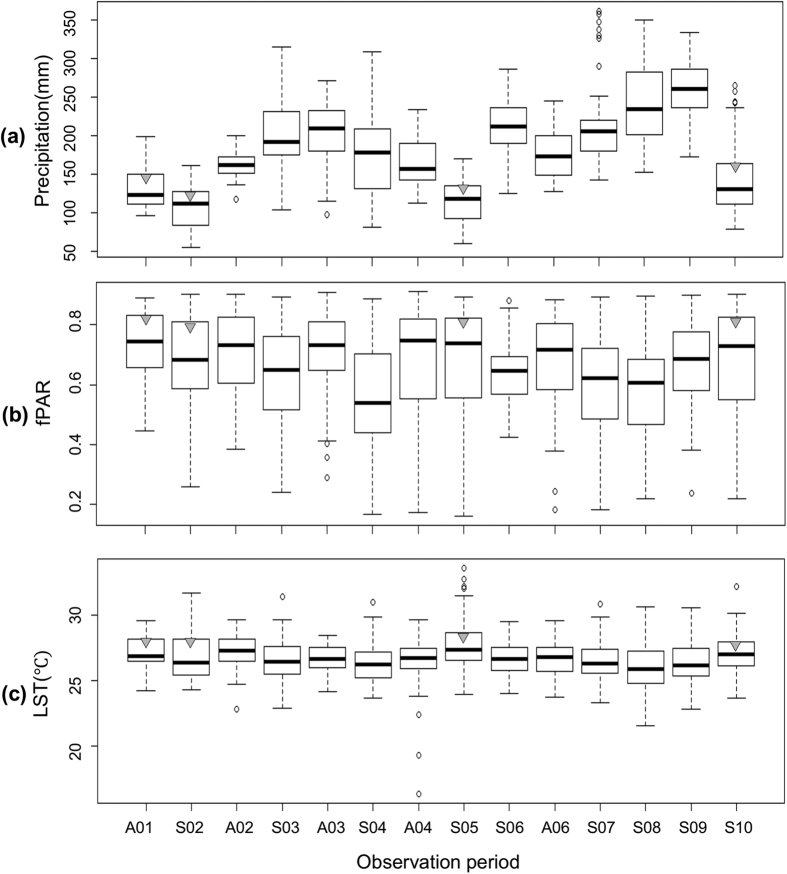
Timeline comparison of forest climatic conditions between large and local-scale GF: (**a**) precipitation; (**b**) fraction of photosynthetically active radiation (fPAR); and (**c**) land surface temperature (LST). The boxplot with the small triangle indicates large-scale GF.

Precipitation, temperature and fPAR showed significant variations with respect to GF scale. The average precipitation (±SE) before large-scale GF (123.90 ± 2.18) was significantly lower than that before local-scale GF (202.14 ± 1.96), (z = −29.7, p < 0.001, generalized linear mixed model (GLMM); Fig. 2a). Three-month average fPAR was significantly higher before large-scale GF (0.69 ± 0.01) than before local-scale GF (0.65 ± 0.01) (z = 1840.7, p < 0.001, GLMM; Fig. 2b). However, LST was not significantly different between large- (27.28 ± 0.10) and local-scale GF (26.56 ± 0.06) (z = 69.4, p < 0.001, GLMM; Fig. 2c).

### Effects of antecedent climatic conditions on GF magnitude in forests before large-scale GF

**Figure 3:**
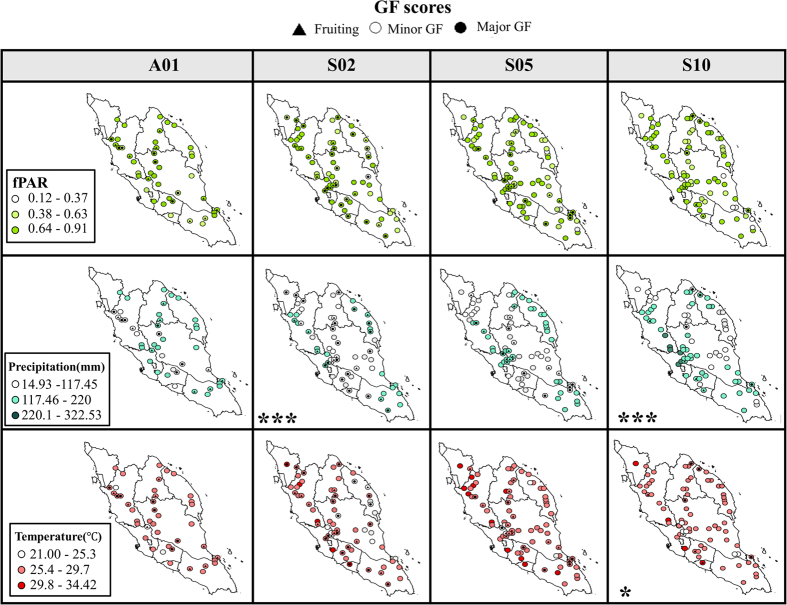
Antecedent climatic conditions (fPAR, precipitation, temperature) variability at forest during large-scale GF events. * and *** indicate significant parameter at p < 0.05 and p < 0.0001 respectively. All maps were developed using ArcGIS v.10 (www.esri.com) and combined using GIMP v.2.8.14 (www.gimp.org).

The generalized linear models (GLMs) showed that precipitation was the best predictor of GF intensity for every large-scale GF. The GLMs suggest that precipitation negatively affected the GF intensity of each forest significantly affected in two GF events, S02 and S10 (Table 1) with almost significant estimate shown for S05. Lowest precipitation has been showed in all large-scale GF events at forest with minor and major flowering (Fig. 3). LST was also a significantly negative predictor in S10 (Table 1) although the lowest preceding temperature was not showed at fruiting or flowering forest (Fig. 3). However, fPAR was not a significant predictor of GF magnitude in any of the four large-scale GFs (Table 1). This was showed as the trend for fPAR was scattered similarly for all type of forests regardless of fruiting and flowering (Fig. 3).

## Discussion

Levels of fPAR and precipitation differed between large- and smaller-scale GF events, but not for LST. Low precipitation, defined as a 3-month average of less than 200 mm, appeared only in the period before large-scale GF events (Fig. 2a), indicating that drought and high photosynthetic radiation are essential in triggering large-scale GF. This result is consistent with previous studies^5^,^6^,^12^,^13^,^14^,^15^,^17^, suggesting that GF events could be triggered by prolonged droughts. However, our results also imply that the relationship between GF and drought may be complex, as prolonged drought may be more effective as a trigger if followed^25^ or preceded^12^ by a warm, prolonged rainy period.

On the other hand, fPAR before large-scale GF was significantly higher than before local-scale GF events. However, high fPAR may not be the primary factor triggering large-scale GF, since variations in fPAR were generally similar among seasons (Fig. 2b). fPAR, which estimates the amount of sunlight absorbed by leaves, may relate to resource accumulation in trees as it is linked to factors related to plant growth, photosynthetic activity, and vegetation cover^26^. Strong solar radiation is also suggested related to higher photosynthesis active radiation in dominant canopy trees besides floral induction^10^,^27^. The magnitude of GF may be influenced by the level of accumulated resources in addition to the intensity of the trigger^9^,^12^. Therefore, high fPAR may indirectly enhance large GF events through resource accumulation in trees, but further studies are needed to examine how high fPAR relates to large-scale GF.

Antecedent LST was also different between large- and smaller-scale GF. The highest average LST occurred before event S05 (Fig. 2c). Over the past two decades, low air temperature has been considered a plausible trigger^2^,^4^,^6^, but average air temperature did not explain the spatial extent of GF in our study. However, we analyzed daytime surface temperature, so our result may not be consistent with those of previous studies. Further studies using daily minimum and maximum fluctuations in LST may help us to understand the impact of temperature on GF.

Forests with low precipitation tended to show high GF scores before GF events, though marginal fixed effects of precipitation were found for large- (A01, S05) (Table 1) and local-scale events (S04, A04) (Table S3). Therefore, we suggest that the spatial extent of drought may be related to that of GF forests^6^. In contrast, we found significant negative effects of LST on GF scores in S10, suggesting that the spatial pattern of LST could also be related to that of GF occurrence (Table 1). What drives these differing climatic effects on the spatial extent of GF forests? In addition to the climatic elements, geographical and latitudinal differences in forest species composition may play a role: for example, forests differ in elevation^6^, exposure to sunlight, and other characteristics. Higher root densities may also affect the flow of water from precipitation through the soil^28^. Considering soil water content by using soil moisture data from radar observations may also help to understand water stress effects on GF^29^. Stimulus strength also determines flowering intensity to differing degrees in different species^30^. Although the Dipterocarpaceae are commonly associated with GF, flowering periods and phenologies do vary among dipterocarp species^3^,^13^,^14^. Thus, we suggest that identifying species in GF is important to understanding the climatic triggers of the phenomenon in this family.

Our remotely-sensed evaluation of climatic variables yielded results consistent with previous studies that used data from meteorological stations. The use of satellite data to study phenology at the landscape and global scales is particularly important for studies of global environmental change^31^,^32^. A remote sensing approach to studying phenology will be especially powerful where meteorological stations are not available. However, there are challenges to using meteorological information estimated by remote sensing. For example, fPAR can be affected by sunlight; it was found to be especially high in the Amazon during drought due to the cloudless sky and high temperatures^33^. By associating LST and fPAR, surface temperatures at the canopy level can be efficiently described, since LST is more related to actual temperature than air temperature^34^. This supports our use of LST to represent surface temperature at the canopy and ground levels.

The potential uncertainty of remote sensing observations should also be borne in mind—for example, there may be atmospheric or surface emissivity effects related to view angle^35^. In the case of LST retrieval, air temperature appeared to be strongly affected by errors^22^, and MOD11A1 data were acquired and processed to correct for low-quality pixels caused by undetected cloud contamination, optical leak correction, and quality flags. The potential uncertainty of estimating flowering or other phenological events using existing remote sensing methods is well understood, but remotely-sensed observations of forest dynamics may still be misunderstood when they contradict ground measurements^36^. Further investigations are needed to cross-validate information gathered from satellites and at ground level to minimize this uncertainty.

We found that drought was the key trigger of large-scale GF events, and that the level of drought may correlate with the magnitude of GF in the dipterocarp forests of Peninsular Malaysia. Since our findings were consistent with those of previous studies, we conclude that a remote sensing approach is useful for phenological studies. Especially since our study had also shown new significant relationship with preceding lower precipitation and lower temperature which is different than previous study^6^,^37^. However, further studies are needed to understand the effects of climatic conditions on GF, as photosynthesis activites and temperature may play indirect roles. Geographical patterns, soil characteristics, and species distribution could all affect the magnitude of plant responses to the climatic conditions, and future investigations of these factors will help us understand how external triggers such as precipitation, light-based photosynthetic activity, and temperature, are related to GF occurrence.

## Materials and methods

### Study area

**Figure 4:**
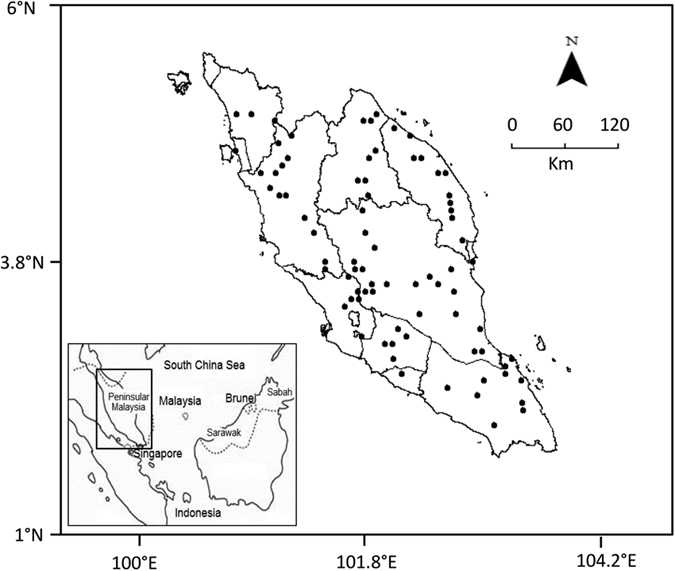
General flowering (GF) observation points in Peninsular Malaysia. The inset map shows the location of the study area in the broader region. All maps were developed using ArcGIS v.10 (www.esri.com) and combined using GIMP v.2.8.14 (www.gimp.org).

The study was conducted in 52–84 forest reserves and public parks (an average of 75 sites each season), scattered throughout Peninsular Malaysia (latitude 100.4° N–103.9° N; longitude 1.6° E–5.8° E; altitude 0–742 m above sea level) over 10 years (Fig. 4, Table S2). Dipterocarps, one of the most important groups in GF studies^1^,^5^,^38^, comprised the dominant tree group at all locations.

### GF observation

To determine the extent of the GF phenomenon, we surveyed the density of fruiting dipterocarp trees in vehicle-accessible forest reserves, protected forests, and old secondary forests from the lowlands to hills^4^,^5^,^6^. We established 52–84 observation points for our surveys, recording the latitude, longitude, and elevation of each on a GPS receiver. Observation points did not necessarily correspond with forest locations, as some observations were made with binoculars from up to 1 km away. Spring and autumn have been suggested as the most probable GF periods in Peninsular Malaysia based on annual precipitation patterns and low temperatures related to monsoon activity^5^. Dipterocarps flower within 2–3 weeks of synchronously dispersing their fruit^39^. Therefore, during spring and autumn GF occurrences, conspicuous red and light-yellowish immature fruits should be visible in the forest canopy. We observed the fruiting status of focal forests twice a year in July (for spring GF) and December (for autumn GF)^4^,^5^,^6^. The fruiting intensity of each forest was classified into four categories as follows: (1) ‘None’—no trees fruiting; (2) ‘Fruiting’—a few trees fruiting; (3) ‘Minor’—more than a few but less than 50% of trees fruiting; and (4) ‘Major’—more than 50% of trees fruiting. To evaluate the spatial extent of GF over Peninsular Malaysia during each putative fruiting season (i.e., spring and autumn), we classified the seasons into two types: (a) large-scale GF when more than 25% of observed forests were classified as minor or major; and (b) local-scale GF when less than 25% of forests were so classified.

### Satellite observations

The two putative periods for spring and autumn GF are estimated as January–March and June–August^5^,^6^. These periods were selected due to the climatic cues expected to occur a few months before GF^5^. For this study, data for three consecutive months were extracted and averaged to estimate the climatic factors preceding the respective GF periods at each forest location. We used a 1-km resolution and raster-type tiles.

### TRMM precipitation observations

To detect drought, we estimated precipitation using TRMM Multi-Satellite Precipitation Analysis (TMPA). TMPA (3B43) version 7 is a combination of several precipitation estimates from multiple satellites with various types of sensors and provides accurate precipitation values, using rain gauge analysis when possible^40^. The sensors include the special sensor microwave/imager (SSMI) and Advanced Microwave Scanning Radiometer - Earth Observing System (AMSR-E), along with the Advanced Microwave Sounding Unit (AMSU) for calibration^41^. Monthly data are stored in individual files with standard self-documenting HDF metadata and distributed via the Internet. Lastly, the monthly gauged values are adjusted in combination with rain-gauged estimates using inverse error variance weighting (TRMM, Global Space Flight Center).

We expected 10 years of monthly observations of precipitation patterns to show distinct normal and dry periods. Since the definition of drought varies among GF studies^6^, we defined drought as three continuous months with less than 200 mm precipitation^42^. Precipitation in Peninsular Malaysia experienced extreme highs and lows during El-Niño Southern Oscillation (ENSO) events^12^,^43^,^44^. With precipitation considered an important spacing mechanism for some tropical plants that triggers anthesis and promotes flowering^45^, remote sensing allows temporal and spatial patterns in precipitation to be identified and associated with GF occurrence.

### MODIS

MODIS is a sensor mounted on a Terra satellite. It is processed into atmosphere, land, cryospheric, and ocean products after acquisition^21^. Here, we used two land data products associated with global change and natural resource monitoring studies^46^. MODIS data were extracted based on land product types: (a) fPAR and (b) LST.

To examine temperature at each site, we used the MODIS LST product, MOD11A1, as it offers daily observational data through 31 and 32 channels, and represents the emissivity of the relative land temperature^47^. The valid range for this daytime land surface temperature variable is 7,500–65,535, using Kelvin units with a 0.02 scale factor. Defined by radiation emitted from the canopy in vegetated areas or the soil surface in bare areas, LST was observed at instant viewing angles^48^. This product represents key land-surface process parameters that combine all surface-atmosphere interactions and energy fluxes between the atmosphere and ground^49^. The basic purpose of the algorithm is to retrieve the temperature value^50^, which is often used in environmental and climatological studies^51^,^52^.

To examine radiation available for photosynthesis, we used the fPAR of MOD15A2 generated from surface reflectance and biome land cover. The incidence of PAR is related to the Radiative Transfer Model, wherein the reflectance of different land covers (biomes) affects the amount of fPAR^36^. fPAR is the fraction of PAR absorbed by the plant canopy for photosynthesis and growth in the 0.4–0.7 μm spectral range. The variable has a range of 0–100, using 0.01 as a scale factor. Used at 8-day intervals, this percentage variable is a good indicator of energy absorbed by vegetation and subsequent carbon uptake in relation to light use efficiency^53^. Solar radiation increases with the duration of sunshine during the dry season, which may trigger GF through resource accumulation^9^,^10^. fPAR is used in studies of ecosystem productivity and for global models of climate and ecology^54^.

### Satellite data processing

Three products are derived from the satellite remote sensing data, comprising MODIS and TRMM satellite data products. The MODIS products include LST (MOD11A1) and fPAR (MOD15A2), both georeferenced in the Universal Transverse Mercator (UTM) coordinate system at a 1-km spatial resolution. By contrast, the TRMM precipitation dataset is TRMM-3B43v7, georeferenced at 0.25 × 0.25 degrees (equivalent to 25 × 25 km), and retrieved from the Goddard East Sciences Data and Information Services Center (https: http://mirador.gsfc.nasa.gov).

All MODIS data were downloaded from the Land Processes Distributed Active Archive Center of the United States Geological Survey (https://lpdaac.usgs.gov), and had the same geometrical properties and geographical coverage. However, the TRMM-3B43v7 dataset had to be georeferenced to the MODIS data. This was accomplished by a geometric correction of the TRMM-3B43v7 data using the ENVI digital image processing system at the Geoscience & Digital Earth Centre (INSTeG), Universiti Teknologi, Malaysia. We used an image-to-image registration approach to transform the TRMM data into the correct geometry, followed by a resampling process wherein the final output was downscaled to a 1 × 1-km grid, equivalent to the MODIS data products.

Observed locations of GF determined by GPS surveys, and all observations of GF in the respective forests, were then computed based on GPS coordinates, with input distances and bearings recorded using the range finder in the binoculars used for identifying all GF points. The GF areas were estimated to an accuracy of 5 ha^5^, as determined by the specifications of the GPS receiver we used.

### Data analysis

Based on the 12 months preliminary observation from 2001 to 2010 where distinct pattern of climatic conditions had been shown in January-March and June-August, this suggest us to observe the climatic variables at each forest over 3-month periods to represent the magnitude of local climatic conditions during putative periods before GF events, a condition that was also found in previous study^5^. Before examining the relationship between climatic condition and GF occurrences (irrespective of scale), we detected and removed outliers using the Shapiro-Wilk test. The validity of the values was confirmed based on the ranges provided in the MODIS Products User’s Guide. We tested the first hypothesis using GLMM^55^. We used GLMMs to identify differences in the three preceding climatic conditions between large- and local-scale GF temporally. The response variable of the model was binary (large or local scale GF) using 3-month averages of precipitation, LST, and fPAR as the fixed effects (the respective months refer to the putative period’s seasonality). The response was still considered as large scale GF although the particular forest was not flowering nor fruiting due to the temporal variability that was considered in the model. Forest ID and seasonality (Autumn, Spring) were used as the random effect, since we observed the same forests for different events and type of seasonality.

For the second hypothesis, we used GLMs to examine the effects of different climatic conditions on fruiting magnitude. This hypothesis was tested only for large-scale GF events, as local-scale GF events showed insufficient variation in fruiting. Fixed effects included 3-month averages of precipitation, LST, and fPAR. As the response variable, we created binary data wherein ‘None’ and ‘Fruiting’ forests were taken as ‘0’ while ‘major’ and ‘minor’ fruiting forests were combined as ‘1’. All analyses were conducted using R software^56^. GLMMs and GLMs were conducted using the glmer() and glm() functions, respectively, from the lme4 package^57^ with identity functions and binomial error distributions. We tested the significance of randomized effects using likelihood ratios^55^ and the Akaike information criterion (AIC).

## Additional Information

**How to cite this article**: Azmy, M. M. *et al*. Satellite-based characterization of climatic conditions before large-scale general flowering events in Peninsular Malaysia. *Sci. Rep.* **6**, 32329; doi: 10.1038/srep32329 (2016).

## Supplementary Material

Supplementary Information

## Figures and Tables

**Table 1:** Generalized linear model (GLM) analysis of binomial error distribution.

GF events	Parameters	n	Estimate	Std. error	z	Pr (>|z|)
A01	Precipitation	44	−0.021	0.012	−1.727	0.084
(0 = 23, 1 = 21)	fPAR		1.625	2.836	0.573	0.567
	LST		−0.001	0.247	−0.005	0.996
S02	Precipitation	64	−0.031	0.012	−2.689	0.007
(0 = 27, 1 = 37)	fPAR		−0.838	1.793	−0.467	0.640
	LST		0.044	0.157	0.283	0.778
S05	Precipitation	82	−0.018	0.009	−1.944	0.052
(0 = 44, 1 = 38)	fPAR		−0.416	1.405	−0.296	0.767
	LST		−0.221	0.134	−1.653	0.098
S10	Precipitation	80	−0.027	0.010	−2.767	0.006
(0 = 59, 1 = 21)	fPAR		0.302	1.728	0.175	0.861
	LST		−0.491	0.237	−2.070	0.038

GF, general flowering; fPAR, photosynthetically active radiation; LST, land surface temperature.
