# Supplementary material for: Satellite-based characterization of climatic conditions before large-scale general flowering events in Peninsular Malaysia
Source: Sci Rep. 2016 Aug 26;6:32329. doi: 10.1038/srep32329 (PMC4999821; doi:10.1038/srep32329)
Supplement: Supplementary Information [file srep32329-s1.pdf]

Title: Satellite-based characterization of climatic conditions before large-scale general flowering events in Peninsular Malaysia

Authors: Muna Maryam Azmy, Mazlan Hashim, Shinya Numata, Tetsuro Hosaka, Nur Supardi Md. Noor, Christine Fletcher

Table S1 General flowering (GF) seasonality, survey occurrence, and scale

Year	Season	Code	GF survey	GF scale
2001	Spring	S01	No	-
2001	Autumn	A01	Yes	Large
2002	Spring	S02	Yes	Large
2002	Autumn	A02	Yes	Local
2003	Spring	S03	Yes	Local
2003	Autumn	A03	Yes	Local
2004	Spring	S04	Yes	Local
2004	Autumn	A04	Yes	Local
2005	Spring	S05	Yes	Large
2005	Autumn	A05	No	-
2006	Spring	S06	Yes	Local
2006	Autumn	A06	Yes	Local
2007	Spring	S07	Yes	Local
2007	Autumn	A07	No	-
2008	Spring	S08	Yes	Local
2008	Autumn	A08	No	-
2009	Spring	S09	Yes	Local
2009	Autumn	A09	No	-
2010	Spring	S10	Yes	Large
2010	Autumn	A10	No	-

Table S 2 Summary of forest characteristics

ID	Y (Degree)	X (Degree)	Height (m)	ID	Y (Degree)	X (Degree)	Height (m)	ID	Y (Degree)	X (Degree)	Height (m)
P2	3.2	101.6	77	P44	4.4	101.2	156	P86	4.6	103.2	42
P3	2.8	101.8	45	P45	4.7	101.0	268	P87	4.7	103.2	24
P4	2.7	102.1	214	P46	4.7	100.9	76	P88	5.0	103.2	30
P5	2.7	102.2	114	P47	4.8	100.8	98	P89	5.0	103.1	45
P7	2.9	102.3	66	P48	4.8	100.8	39	P90	5.2	102.9	37
P10	2.8	102.4	82	P50	5.0	100.7	27	P91	5.2	102.8	130
P12	3.1	102.6	42	P53	5.8	100.7	31	P92	5.5	102.8	14
P13	3.1	103.1	30	P55	5.7	101.0	337	P94	5.6	102.6	15
P14	2.9	103.4	7	P56	5.5	101.2	445	P95	5.6	102.6	32
P16	2.6	103.4	30	P59	5.4	101.0	172	P97	5.8	102.4	35
P18	2.6	103.3	45	P60	5.2	101.1	95	P99	5.7	102.3	63
P19	2.5	103.8	13	P61	5.1	101.0	93	P100	5.7	102.2	49
P21	2.3	103.7	23	P62	5.0	100.9	79	P102	5.3	102.3	44
P22	2.2	103.9	34	P63	3.3	101.7	152	P103	5.3	102.3	48
P24	1.9	103.9	15	P65	3.3	101.8	542	P105	5.2	102.2	78
P27	1.8	103.9	33	P66	3.4	101.8	551	P107	4.9	102.1	134
P28	1.6	103.5	62	P67	3.4	101.9	151	P108	4.9	102.0	105
P32	2.1	102.9	80	P68	3.5	102.0	88	P109	4.7	102.1	166
P33	2.2	103.4	59	P69	3.4	102.0	67	P110	4.5	102.0	152
P34	2.3	102.3	46	P72	3.5	102.2	62	P111	4.5	102.0	237
P35	2.5	102.2	82	P73	3.5	102.6	59	P112	4.2	102.0	145
P36	3.6	101.7	248	P74	3.6	102.8	78	P113	4.2	102.0	151
P38	3.6	101.7	742	P76	3.5	102.9	55	P115	4.0	102.1	119
P40	3.7	101.4	63	P78	3.4	103.1	29	P117	3.8	101.8	168
P42	3.8	101.4	46	P79	3.7	103.1	49	P119	3.7	101.8	460
P43	4.2	101.3	114	P83	4.4	103.2	47	P120	3.7	101.9	140
P44	4.4	101.2	156	P85	4.5	103.2	48				

Table S 3 GLM analysis of binomial error distribution for local-scale GF episodes

GF events	Parameters	n	Estimate	Std. Error	z	Pr(>|z|)
A02 (0=59)	Precipitation	59	-1.248e-16	2.753e+03	0	1
	fPAR		4.141e-14	3.516e+05	0	1
	LST		2.759e-16	3.560e+04	0	1
A03 (0=61, 1=4)	Precipitation	65	7.821e-04	1.685e-02	0.046	0.963
	fPAR		8.860e+00	6.791e+00	1.305	0.192
	LST		2.303e-01	5.499e-01	0.419	0.675
A04 (0=70, 1=4	Precipitation	74	-0.05556	0.02796	-1.987	0.0469
	fPAR		8.91109	6.64581	1.341	0.1800
	LST		-0.54167	0.37146	-1.458	0.1448
A06 (0=80)	Precipitation	80	8.684e-19	1.233e+03	0	1
	fPAR		-1.687e-16	2.673e+05	0	1
	LST		1.799e-17	3.287e+04	0	1
S03 (0=60)	Precipitation	60	6.876e-18	1.174e+03	0	1
	fPAR		4.977e-15	2.957e+05	0	1
	LST		1.595e-15	3.104e+04	0	1
S04 (0=64, 1=7)	Precipitation	71	0.02548	0.01031	2.472	0.0134
	fPAR		-3.81593	2.59443	-1.471	0.1413
	LST		0.14934	0.30783	0.485	0.6276
S06 (0=54, 1=1)	Precipitation	55	2.132	865.179	0.002	0.998
	fPAR		1195.551	467272.450	0.003	0.998
	LST		-4.071	7335.965	-0.001	1.000
S07 (0=78,1=1)	Precipitation	79	0.004523	0.021910	0.206	0.836
	fPAR		11.624342	11.849746	0.981	0.327
	LST		-0.131834	0.727413	-0.181	0.856
S08 (0=79)	Precipitation	79	-1.480e-18	8.209e+02	0	1
	fPAR		4.144e-15	2.531e+05	0	1
	LST		-9.673e-16	2.043e+04	0	1
S09 (0=77, 1=1)	Precipitation	78	-0.05897	0.05483	-1.076	0.282
	fPAR		-1.68590	10.86750	-0.155	0.877
	LST		-0.43544	0.90906	-0.479	0.632

GF, general flowering; fPAR, photosynthetically active radiation; LST, land surface temperature
